# Effect of vaginal delivery history on oliceridine ED₅₀ combined with propofol during painless hysteroscopy: a modified Dixon sequential study

**DOI:** 10.3389/fmed.2026.1870477

**Published:** 2026-08-17

**Authors:** Xinyang Zhang, Xiaoyu Gu, Jinfeng Cao, Jianlong Du, Liuqin Jiang

**Affiliations:** Department of Anesthesiology, No.215 Hospital of Shaanxi Nuclear Industry, Xianyang, Shaanxi, China

**Keywords:** body movement, median effective dose (ED_50_), modified Dixon sequential method, oliceridine, painless hysteroscopy, propofol, vaginal delivery history

## Abstract

**Objective:**

To determine the median effective dose (ED_50_) of oliceridine combined with propofol for inhibiting movement response during hysteroscopy in patients with versus without a history of vaginal delivery.

**Methods:**

Patients scheduled for hysteroscopy who met the inclusion criteria were stratified into two cohorts: those with no history of vaginal delivery (NVD cohort, including nulliparous women and those with prior cesarean section only) and those with a history of vaginal delivery (VD cohort). Based on the Dixon sequential outcomes, patients were further classified as negative subcohort (successful hysteroscopy, indicating no body movement) or positive subcohort (failed hysteroscopy, indicating body movement). An initial intravenous oliceridine dose of 20 μg/kg was administered, followed by propofol 1.5 mg/kg after 2 min. Successful induction was defined as a Modified Observer’s Assessment of Alertness/Sedation (MOAA/S) score of 0 (no response to trapezius squeeze) and a bispectral index (BIS) < 60. Intraoperative propofol boluses (0.5 mg/kg) maintained BIS between 40 and 60. Oliceridine dosing was adjusted using a modified Dixon up-and-down sequential method (dose ratio 1:1.2 between adjacent levels) until at least seven crossover points were recorded. The primary outcome was the ED_50_ of oliceridine for suppressing body movement, calculated by probit regression with 95% confidence interval (CI). Cohort comparison of ED_50_ was performed using a *Z*-test.

**Results:**

The ED_50_ of oliceridine for suppressing movement response during hysteroscopy was 21.603 μg/kg (95% CI: 19.922–23.752) in the NVD cohort and 17.816 μg/kg (95% CI: 15.880–19.981) in the VD cohort. A formal *Z*-test confirmed that the NVD cohort required a 21% higher ED_50_ than the VD cohort, and this difference was significant (*Z* = 2.646, *p* = 0.008). No significant baseline differences were observed between the negative and positive subcohorts (all *p* > 0.05). The NVD negative subcohort required higher oliceridine (*Z* = 3.19, *p* < 0.001); the VD negative subcohort consumed more propofol (*t* = 2.11, *p* = 0.038) and oliceridine (*Z* = 3.05, *p* = 0.002). No severe adverse events occurred.

**Conclusion:**

During painless hysteroscopy, the ED_50_ of oliceridine combined with propofol for suppressing movement response was 21% higher in patients without a history of vaginal delivery than in those with such a history. This finding suggests that a history of vaginal delivery may be a useful factor to consider when individualizing initial oliceridine dosing in this setting.

**Clinical trial registration:**

ChiCTR2500095126 (https://www.chictr.org.cn)

## 1 Introduction

Hysteroscopy uses a distension medium to expand the uterine cavity, enabling direct visualization of the cervical canal, internal cervical os, uterine cavity, and tubal ostia via a fiberoptic hysteroscope. This minimally invasive technique allows precise diagnosis, targeted biopsy, and therapeutic interventions for intrauterine pathologies. Hysteroscopy is regarded as the gold standard for diagnosing and treating intrauterine disorders (1). It is increasingly performed as an ambulatory procedure due to its high safety profile, cost-effectiveness, and patient satisfaction (2). However, cervical dilation and endometrial manipulation often cause intolerable pain without adequate sedation and analgesia, leading to procedure abandonment or incomplete examination (3). In China, moderate sedation with rapid-onset, short-acting agents is preferred for ambulatory hysteroscopy to minimize cardiorespiratory depression (4).

Propofol, a short-acting sedative, is widely used for endoscopic procedures due to its rapid onset and recovery (5). Owing to limitations such as injection pain, respiratory depression, hypotension, and bradycardia, the administration of adjunctive analgesics is mandated to mitigate these side effects (6). Current total intravenous anesthesia (TIVA) protocols combining propofol with opioids (e.g., sufentanil) aim to balance efficacy and hemodynamic stability. However, sufentanil is associated with adverse effects, including cough, respiratory depression, postoperative nausea and vomiting (PONV), and hemodynamic instability (7), which underscores the necessity for safer alternatives.

Oliceridine is a biased *μ*-opioid receptor agonist that preferentially activates G-protein signaling over *β*-arrestin pathways. This selective bias is clinically relevant because it correlates with fewer episodes of respiratory depression and PONV (8). This pharmacological profile establishes oliceridine as a highly suitable candidate for hysteroscopy, a procedure requiring rapid onset of analgesia, minimal cardiorespiratory effects, and prompt recovery. However, few hysteroscopy dose-finding studies have stratified patients by vaginal delivery history, a variable that may fundamentally alter cervical mechanosensitivity. Vaginal delivery induces permanent structural and neural remodeling of the cervix: increased compliance resulting from extracellular matrix reorganization and collagen remodeling (9), and altered mechanosensitivity due to increased excitability of cervical mechanosensitive afferents (10). To date, no study has reported delivery history-stratified the median effective dose (ED_50_) and 95% effective dose (ED_95_) values for oliceridine in hysteroscopy, leaving clinicians without evidence-based dosing guidance for this common patient characteristic. We therefore hypothesized that nulliparous patients require higher oliceridine doses than parous patients to suppress movement responses during hysteroscopy. Previous dose-finding studies have largely overlooked the clinically relevant heterogeneity in opioid requirements. To test this hypothesis and address this gap, this trial determined the ED_50_ of oliceridine combined with propofol separately for patients with and without a history of vaginal delivery. Our goal is to provide stratified, ready-to-use dosing references for precision anesthesia in ambulatory hysteroscopy.

## 2 Methods

### 2.1 Ethics and trial registration

The trial was conducted at Shaanxi Nuclear Industry 215 Hospital. All participants provided written informed consent and were free to withdraw from the trial at any time. The study was approved by the Institutional Review Board of the Ethics Committee of NO.215 Hospital of Shaanxi Nuclear Industry (approval number: 2024(031)) and was registered in the Chinese Clinical Trial Registry (ChiCTR2500095126). All procedures performed on the patients adhered to the Declaration of Helsinki and its subsequent amendments.

### 2.2 Study population

#### 2.2.1 Inclusion criteria

(1) Patients aged 18 to 64 years; (2) Body Mass Index (BMI) of 18.5 to 29.9 kg/m^2^; (3) American Society of Anesthesiologists (ASA) Physical Status Classification I to II; (4) Patients diagnosed by gynecologists as requiring hysteroscopic examination.

This BMI range (18.5–29.9 kg/m^2^) follows the WHO classification, which defines BMI < 18.5 as underweight and ≥ 30 as obesity (11). Both propofol and oliceridine are highly lipophilic; obesity expands adipose tissue mass, increasing their volume of distribution and potentially confounding dose-finding estimates (12). Excluding underweight and obese individuals therefore minimizes pharmacokinetic heterogeneity and improves ED_50_/ED_95_ precision.

#### 2.2.2 Exclusion criteria

(1) Patients with a history of severe diseases of the heart, brain, lungs, liver, kidneys, endocrine system, or mental disorders; (2) Heart rate (HR) < 50 beats per minute; (3) History of unresolved acute respiratory infection within 14 days or obvious difficult airway; (4) Patients with known allergy to propofol, oliceridine, or any of their components; (5) A history of alcoholism or long-term use of sedatives or analgesics; (6) Patients requiring special treatment under hysteroscopy.

#### 2.2.3 Withdrawal criteria

(1) Surgical duration > 30 min; (2) Patients who voluntarily request to withdraw from the study; (3) Patients lost to follow-up.

### 2.3 Cohort stratification and modified Dixon sequential method

As prespecified in the trial registration, stratification by vaginal delivery history was planned. Patients were screened according to the inclusion and exclusion criteria and signed the anesthesia informed consent form in advance. Eligible patients were stratified into two cohorts: the cohort with no history of vaginal delivery (NVD cohort, including nulliparous women and those with prior cesarean section only) and the cohort with a history of vaginal delivery (VD cohort). The modified Dixon sequential method (13) was used in both cohorts of this trial, wherein the oliceridine injection dose for each subsequent subject was dynamically adjusted based on hysteroscopic outcomes (success/failure) of the preceding patient (14). Failure was defined as the occurrence of involuntary limb/body movement during the procedure (15). If the hysteroscopic examination failed (namely, a positive response, assigned to the positive subcohort), the dose of oliceridine was increased for the next case. Conversely, if the examination was successful (that is, a negative response, assigned to the negative subcohort), the dose was decreased. The dose ratio of oliceridine between adjacent dose levels was consistently maintained at 1:1.2.

### 2.4 Study interventions

All patients fasted for 8 h and abstained from clear fluids for 2 h preoperatively, with no preoperative medications administered. Upon entering the operating room, routine monitoring including peripheral oxygen saturation (SpO₂), non-invasive blood pressure, and electrocardiography was initiated using an IntelliVue MP5/MP5T/MP5SC patient monitor (Philips, the Netherlands). Patients received oxygen via face mask at 5 L/min, and a thin pillow was placed under the shoulders to prevent upper airway obstruction. Rescue equipment, including a bag-valve mask, oropharyngeal airways, laryngoscopes, and endotracheal intubation kits, was prepared. After establishing an intravenous access, anesthesia induction was performed.

The initial oliceridine dose (Lot: TAG24K02, Jiangsu Enhua Pharmaceutical Co., Ltd.) was established at 20 μg/kg based on pre-trial data and morphine-equivalent conversion, with this regimen demonstrating equipotent analgesic efficacy to 0.1 μg/kg sufentanil. For the first patient in each cohort, 20 μg/kg oliceridine was administered intravenously. After a 2-min interval, 1.5 mg/kg propofol (Lot: 7A250105-2; Guangdong Jiabo Pharmaceutical Co., Ltd.) was infused over 40 s, based on preliminary dose-finding trials and previous studies (14). Successful induction of anesthesia was defined as a Modified Observer’s Assessment of Alertness/Sedation (MOAA/S) (16) score of 0 (no response to trapezius squeeze) combined with a Bispectral Index (BIS) value below 60. Upon meeting these criteria, surgery was initiated with the insertion of the speculum. In case of hysteroscopic failure (as defined in Section 2.3), an immediate rescue bolus of propofol 0.5 mg/kg was administered; persistent movements triggered an additional 0.5 mg/kg bolus to maintain anesthetic depth (BIS 40–60) and ensure patient safety. Surgery resumed only after achieving complete motor quiescence. Intraoperative propofol 0.5 mg/kg boluses were titrated to maintain BIS within 40–60. The formal testing phase commenced at the first crossover point and continued until at least seven crossover points were documented to satisfy pharmacodynamic endpoint criteria.

Postoperatively, all patients were transferred to the post-anesthesia care unit (PACU) and monitored for 6 h. To minimize bias, all anesthetic and surgical procedures were performed by two specialists: a senior anesthesiologist (with over 5 years of experience) and a designated gynecologist.

### 2.5 Sample size estimation

Sample size was not determined by conventional power analysis but followed the modified Dixon sequential design (13). The formal phase started after the first crossover and continued until at least seven crossover points were recorded for each cohort. Because the number of patients required to reach at least seven crossovers depends on individual sequential response patterns, this process dynamically yielded final sample sizes of 30 patients in the VD cohort and 21 patients in the NVD cohort.

### 2.6 Statistical analysis

Data were analyzed with SPSS 27.0 (SPSS Inc.). The normality of continuous variables was assessed using the Shapiro–Wilk test. Normal-distributed data are reported as mean ± SD and compared by *t*-test; non-normal data as median (IQR) and compared using the Mann–Whitney *U* test for independent cohorts. Probit regression was chosen to calculate the ED₅₀ and ED_95_ of oliceridine combined with propofol, along with their 95% confidence intervals (CIs), as it is the established standard for up-and-down sequential dose-finding designs. To formally compare the estimated median ED_50_ of oliceridine combined with propofol between the NVD and VD cohorts, a *Z*-test was conducted utilizing the standard errors derived from their respective 95% CIs. Multiple time-point comparisons used the Friedman test, with significant results (*p* < 0.05) followed by Wilcoxon signed-rank tests, where the threshold of *p* < 0.0125 was a Bonferroni correction applied specifically to account for the four time-point comparisons against baseline (*α* = 0.05/4 = 0.0125) for the hemodynamic parameters. Categorical data are presented as *n* (%) and compared by χ^2^/Fisher’s exact test. No formal power calculation was performed for secondary safety endpoints, as the sample size in the Dixon design is determined by the number of crossover points rather than by conventional power analysis. Statistical significance was set at *p* < 0.05. Sequential response plots and dose–response curves illustrating oliceridine’s efficacy in suppressing intraoperative body movement were generated using GraphPad Prism 10.

## 3 Outcomes

### 3.1 Primary outcomes

The primary outcome was the ED₅₀ of oliceridine for suppressing body movement, calculated by probit regression with 95% CI.

### 3.2 Secondary outcomes

The ED_95_ of oliceridine for suppressing body movement, calculated by probit regression with 95% CI.

*Hemodynamic and anesthetic depth parameters*: HR, mean arterial pressure (MAP), SpO₂, and BIS were recorded at predefined time points: baseline upon room entry (T_0_), immediately after successful anesthesia induction (T_1_), at 1 min (T_2_) and 5 min (T_3_) after surgical initiation, and upon emergence from anesthesia (T_4_).

*Pharmacological and procedural metrics*: specifically, the initial propofol dose, total intraoperative propofol consumption, utilization of vasoactive agents, and total surgical duration.

*Intraoperative adverse events*: body movement, cough, respiratory depression, hypotension, bradycardia, and propofol injection pain were documented, as well as pain intensity upon awakening quantified using the Numeric Rating Scale (NRS).

*Postoperative complications*: nausea, vomiting, abdominal pain, headache, dizziness, respiratory depression, hypotension, and bradycardia, were recorded, along with NRS assessments conducted at 2 h and 6 h postoperatively. Adverse events were managed according to the following predefined protocol. Hypoxemia [defined as SpO₂ < 90% for > 10 s (17)] was treated by increasing oxygen supplementation to 10 L/min and performing a jaw thrust maneuver. Respiratory depression [defined as a respiratory rate < 10 breaths/min (18)] was managed with manual ventilation if it persisted. Bradycardia (HR < 50 beats/min) was treated with intravenous atropine (0.3–0.5 mg). Hypotension [defined as SBP < 90 mmHg, MAP < 60 mmHg, or a > 20% decrease from baseline (19)] was corrected with intravenous dopamine (2 mg).

## 4 Comparison of baseline characteristics and drug consumption between subcohorts

**Figure 1:**
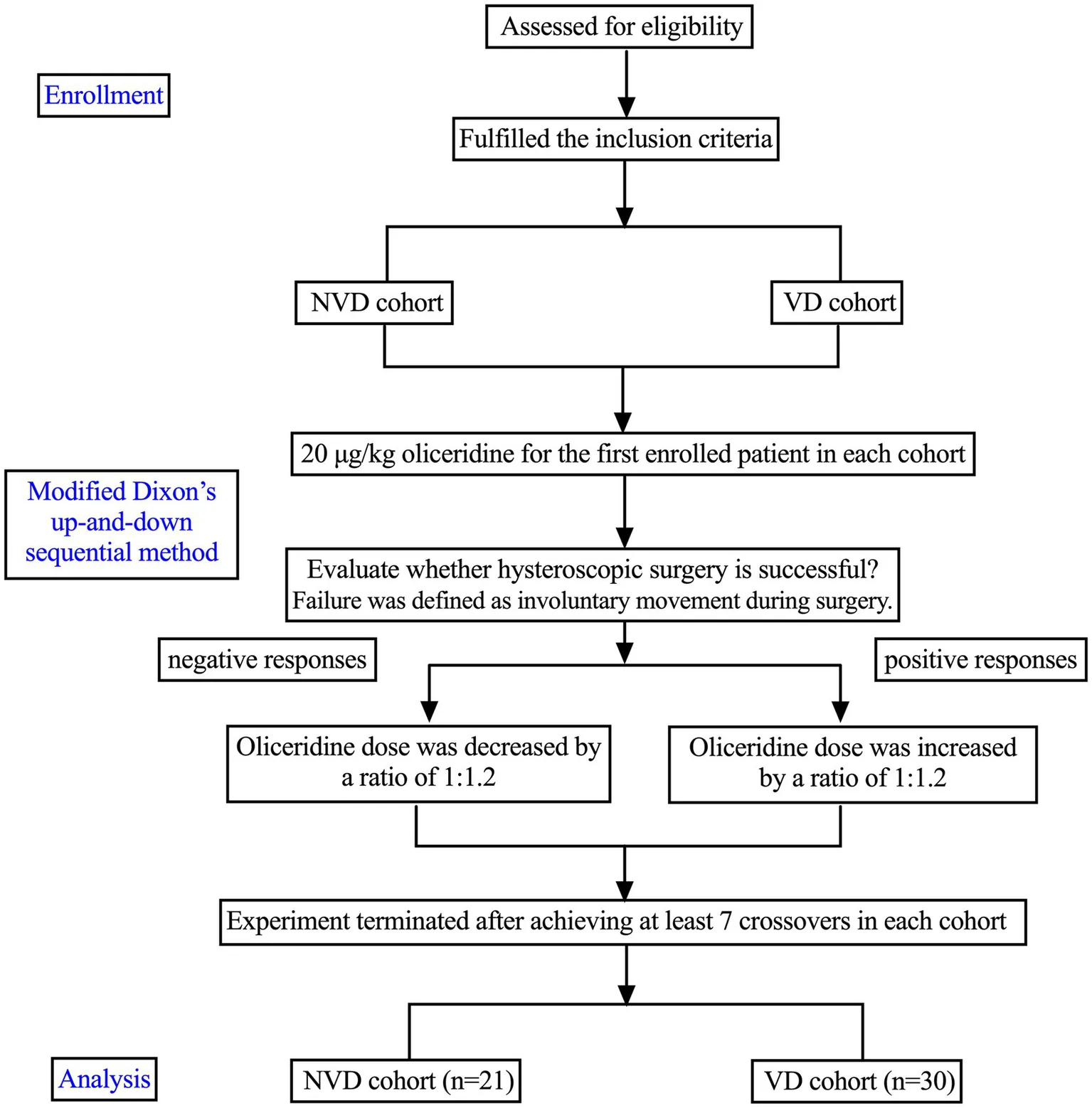
Flow diagram of the study. NVD, non-vaginal delivery; VD, vaginal delivery.

The study employed a modified Dixon’s up-and-down sequential method. The formal testing phase commenced at the first crossover point and continued until at least seven crossover points were obtained. Based on this design, a total of 51 patients were enrolled and subsequently stratified by vaginal delivery history into two cohorts: the VD cohort (*n* = 30) and the NVD cohort (*n* = 21) (Figure 1).

In both cohorts, the negative and positive subcohorts did not differ significantly in baseline characteristics, namely age, BMI, ASA status, history of motion sickness, induction time, surgical duration, and propofol dose (Table 1, *p* > 0.05).

**Table 1:** Baseline demographic and clinical characteristics of the patients.

Characteristic	NVD cohort (*n* = 21)		*t/Z/χ ^2^* value	*p* value	VD cohort (*n* = 30)		*t/Z/χ ^2^* value	*p* value
	Positive	Negative			Positive	Negative		
Age (years)	37.91 (5.22)	41.20 (9.99)	*t* (0.96)	0.35	48.53 (09.88)	46.93 (9.07)	*t* (0.46)	0.65
BMI (kg/m^2^)	24.04 (2.36)	22.92 (3.16)	*t* (0.93)	0.37	23.34 (2.54)	22.97 (2.82)	*t* (0.38)	0.71
ASA								
I	1	0	*χ 2* (0.96)	0.33	0	1	*χ ^2^* (1.03)	0.31
II	10	10			15	14		
Motion sickness	1	3	*χ 2* (1.49)	0.22	1	5	*χ ^2^* (3.33)	0.07
Induction time (min), Median (IQR)	3.00 (1.00)	4.00 (1.00)	*Z* (0.65)	0.52	3.00 (1.00)	3.00 (1.00)	*Z* (0.00)	1.00
Hysteroscope duration (min)	12.73 (7.25)	12.20 (5.20)	*t* (1.63)	0.12	10.00 (7.00)	9.00 (5.00)	*Z* (1.04)	0.30
Induction propofol dosage (mg), Median (IQR)	90.00 (10.00)	85.00 (12.50)	*Z* (1.21)	0.23	90.00 (20.00)	90.00 (20.00)	*Z* (0.50)	0.62
Total propofol dosage (mg), Median (IQR)	200.00 (90)	197.50 (127.5)	*Z* (0.53)	0.60	190.00 (70.00)	150.00 (30.00)	*t* (2.11)	0.04*
Oliceridine dosage (μg), Median (IQR)	20.00 (0.00)	24.00 (1.00)	*Z* (3.19)	0.00*	16.67 (2.78)	20.00 (3.33)	*Z* (3.05)	0.00*

Data are presented as the mean ± SD, median (interquartile range) or as the number (proportion) as appropriate. Two-sided *t*-test, U-text, or Chi-square test, *p < 0.05.

In the NVD cohort, significantly higher oliceridine consumption was documented in the negative subcohort compared to the positive subcohort (*Z* = 3.19, *p* < 0.001). In the VD cohort, both total propofol consumption and oliceridine dosage requirements were significantly higher in the negative subcohort (propofol: *t* = 2.11, *p* = 0.038; oliceridine: *Z* = 3.05, *p* = 0.002).

## 5 Results

### 5.1 Effective doses of oliceridine for intraoperative movement suppression

**Figure 2:**
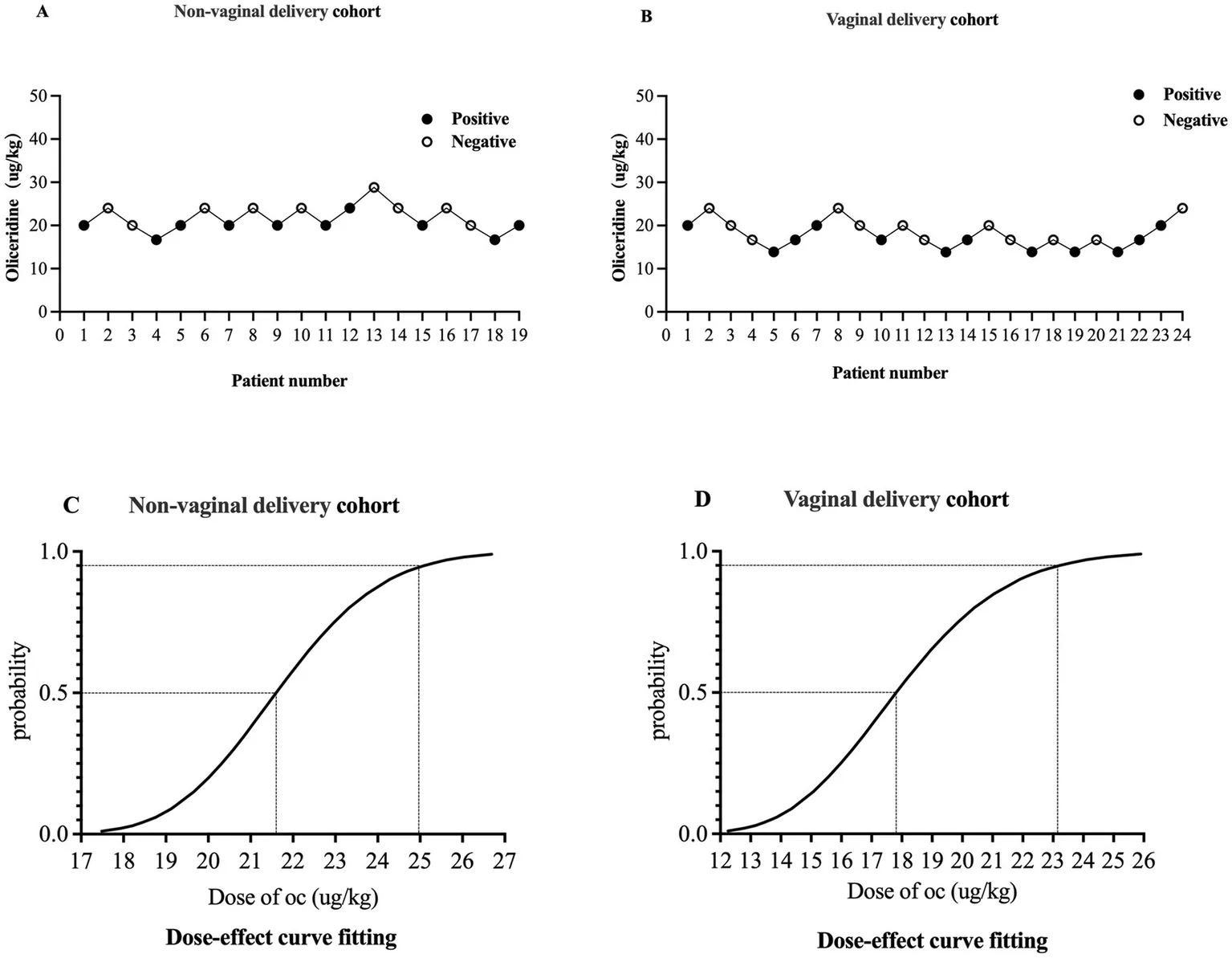
Individual response sequences **(A,B)** and dose-effect curves (C,D) of oliceridine in NVD and VD cohorts. **(A,B)** Individual dosing sequences determined by the modified Dixon up-and-down sequential method in the NVD cohort **(A)** and VD cohort **(B)**. Solid black dots represent positive responses (procedural failure, defined as involuntary movement); open white circles represent negative responses (procedural success without movement). Testing started at an initial oliceridine dose of 20 μg/kg with a 1:1.2 dose adjustment ratio between adjacent levels. Horizontal dashed lines indicate calculated ED_50_ and ED_95_ values. **(C,D)** Dose-effect curves fitted by probit regression for the NVD cohort **(C)** and VD cohort **(D)**, showing the predicted probability of movement suppression across oliceridine doses. Vertical dashed lines mark the estimated ED_50_ and ED_95_ values. ED_50_, median effective dose; ED_95_, 95% effective dose; NVD, non-vaginal delivery; VD, vaginal delivery; oc, oliceridine.

The ED₅₀ of oliceridine combined with propofol for suppressing intraoperative body movement was 21.603 μg/kg (95% CI: 19.922–23.752 μg/kg) in the NVD cohort and 17.816 μg/kg (95% CI: 15.880–19.981 μg/kg) in the VD cohort. Thus, compared to the VD cohort, the NVD cohort required a 21% higher ED₅₀. A formal statistical comparison utilizing a *Z*-test confirmed that this difference in ED₅₀ requirements between the two cohorts was statistically significant (*Z* = 2.646, *p* = 0.008). The ED_95_ was 25.090 μg/kg (95% CI: 23.120–36.414 μg/kg) for NVD and 23.216 μg/kg (95% CI, 20.459–42.365 μg/kg) for VD, with an 8% higher ED_95_. Due to the inherent limitations of the modified Dixon up-and-down method, the ED_95_ estimates should be interpreted as heuristic approximations rather than definitive parameters, and their overlapping 95% CIs preclude any comparative statistical inference. Sequential response plots and dose–response curves further illustrated these findings (Figure 2).

### 5.2 Vital signs of the two cohorts of patients at different time points

**Figure 3:**
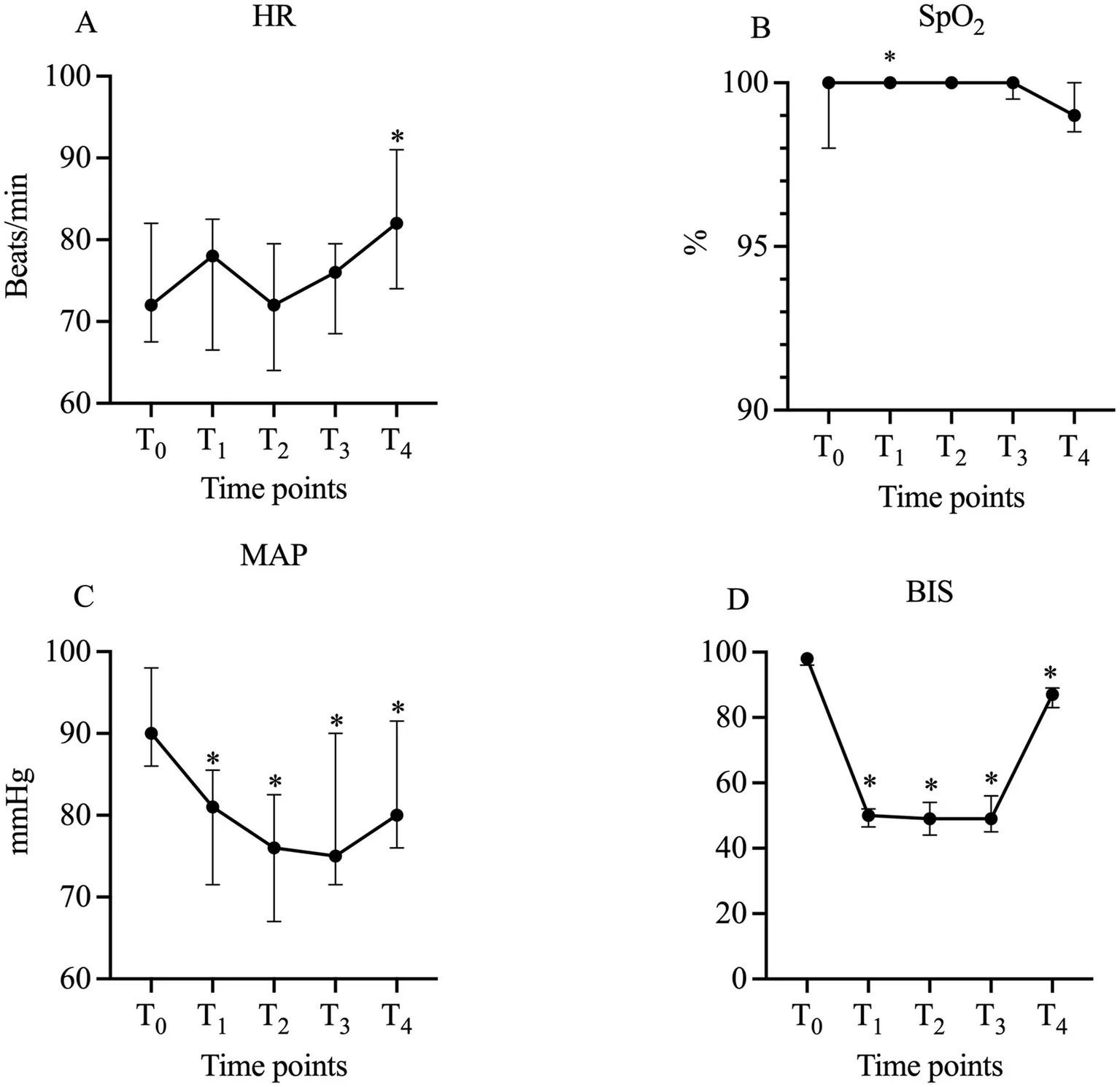
Hemodynamic parameters and anesthetic depth at different time points in the NVD cohort. Serial measurements of HR **(A)**, MAP **(B)**, SpO_2_ **(C)**, and BIS value **(D)** in the NVD cohort (*n* = 21). Data are presented as median, and error bars are presented as IQR (25th–75th percentile). Friedman tests revealed significant overall differences in all parameters (*p* < 0.05). *Post hoc* comparisons between T_0_ and subsequent time points (T_1_–T_4_) were conducted using Wilcoxon signed-rank tests with Bonferroni correction (adjusted *α* = 0.0125). *p < 0.0125 versus T_0_. T_0_, baseline; T_1_, post-induction stabilization; T_2_, 1 min after surgical initiation; T_3_, 5 min after surgical initiation; T_4_, return of consciousness. BIS, bispectral index; HR, heart rate; IQR, interquartile range; MAP, mean arterial pressure; NVD, non-vaginal delivery; SpO_2_, saturation of peripheral oxygen.

In the NVD cohort, compared with T_0_, HR was significantly increased at T_4_ (*p* < 0.0125); SpO₂ was decreased at T_1_ (*p* < 0.0125); MAP was significantly decreased at T_1_, T_2_, T_3_, and T_4_ (*p* < 0.0125); and BIS was significantly decreased at T_1_, T_2_, T_3_, and T_4_ (*p* < 0.0125) (Figure 3).

**Figure 4:**
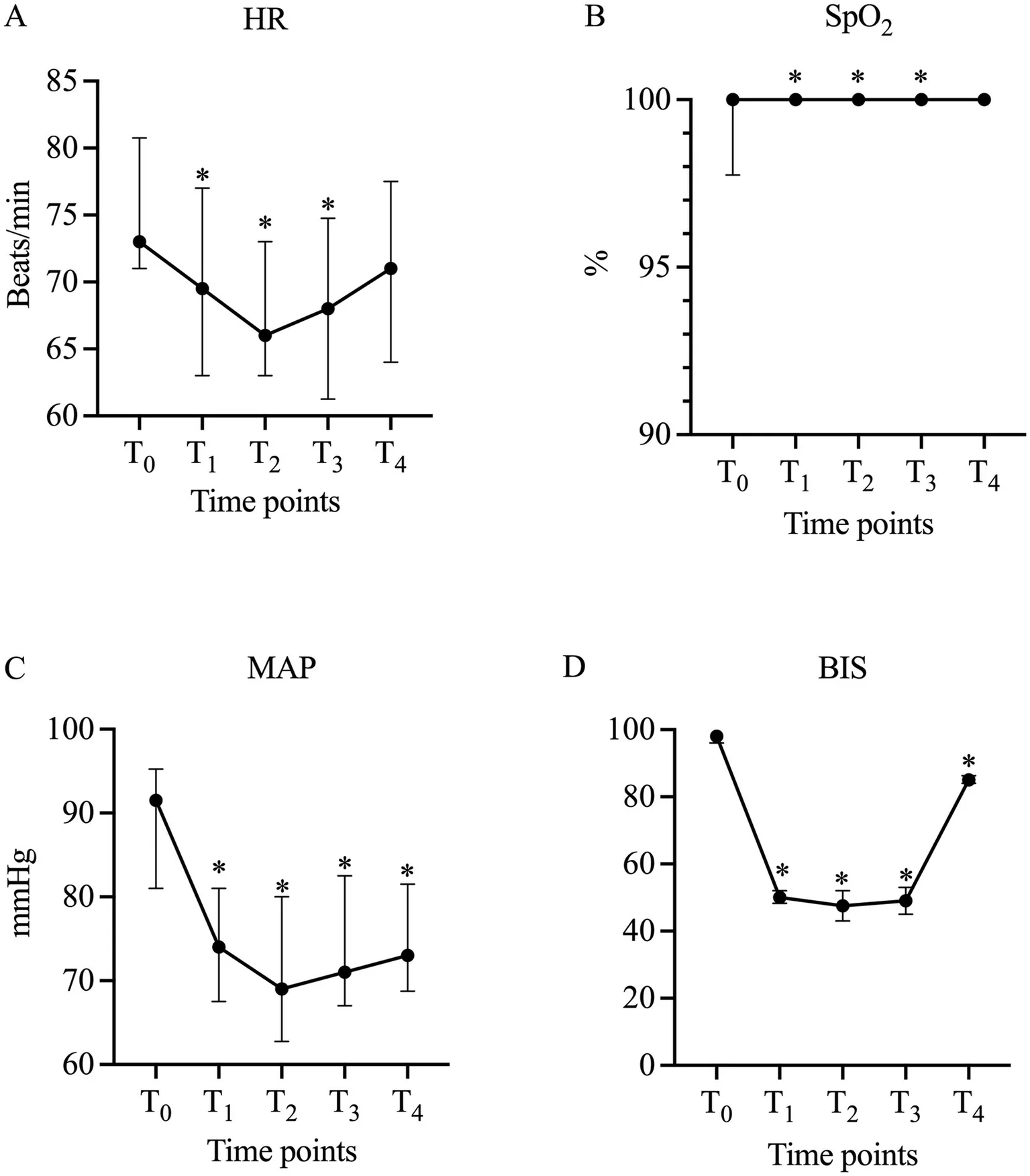
Hemodynamic parameters and anesthetic depth at different time points in the VD cohort. Serial measurements of HR **(A)**, MAP **(B)**, SpO2 **(C)**, and BIS value **(D)** in the VD cohort (*n* = 30). Data are presented as median, and error bars are presented as IQR (25th-75th percentile). Friedman tests revealed significant overall differences in all parameters (*p* < 0.05). Post hoc comparisons between T0 and subsequent time points (T1–T4) were conducted using Wilcoxon signed-rank tests with Bonferroni correction (adjusted *α* = 0.0125). *p < 0.0125 versus T0. T0, baseline; T1, post-induction stabilization; T2, 1 min after surgical initiation; T3, 5 min after surgical initiation; T4, return of consciousness. BIS, bispectral index; HR, heart rate; IQR, interquartile range; MAP, mean arterial pressure; SpO2, saturation of peripheral oxygen; VD, vaginal delivery.

In the VD cohort, compared with T_0_, HR was significantly decreased at T_1_, T_2_, and T_3_ (all *p* < 0.0125); SpO₂ was decreased at T_1_, T_2_, and T_3_ (all *p* < 0.0125); MAP was significantly decreased at T_1_, T_2_, T_3_, and T_4_ (all *p* < 0.0125); and BIS was significantly decreased at T_1_, T_2_, T_3_, and T_4_ (all *p* < 0.0125) (Figure 4).

### 5.3 Intraoperative adverse reactions

In the VD cohort, bradycardia (1/30, 3.33%), propofol injection pain (6/30, 20.00%), hypotension (1/30, 3.33%), and hypoxemia (1/30, 3.33%) were observed; whereas in the NVD cohort, only propofol injection pain (2/21, 9.52%) and hypoxemia (1/21, 4.76%) were reported, with all adverse events being mild in severity (Table 2).

**Table 2:** Intraoperative and postoperative adverse events data.

Adverse events	NVD cohort (*n* = 21)	VD cohort (*n* = 30)
Intraoperative adverse events		
Injection pain	2 (9.52%)	6 (20.00%)
Hypoxemia	1 (4.76%)	1 (3.33%)
Bradycardia	0	1 (3.33%)
Hypotension	0	1 (3.33%)
Postoperative adverse events		
Dizziness	0	1 (3.33%)
Nausea	2 (9.52%)	0
Vomiting	1 (4.76%)	0

Data are presented as *n* (%). Safety data are reported strictly as descriptive monitoring without formal statistical comparisons due to limited sample size.

### 5.4 Postoperative adverse reactions

Dizziness (1/30, 3.33%) was reported in the VD cohort, while nausea (2/21, 9.52%) and vomiting (1/21, 4.76%) occurred in the NVD cohort (Table 2).

### 5.5 Numeric rating scale

**Figure 5:**
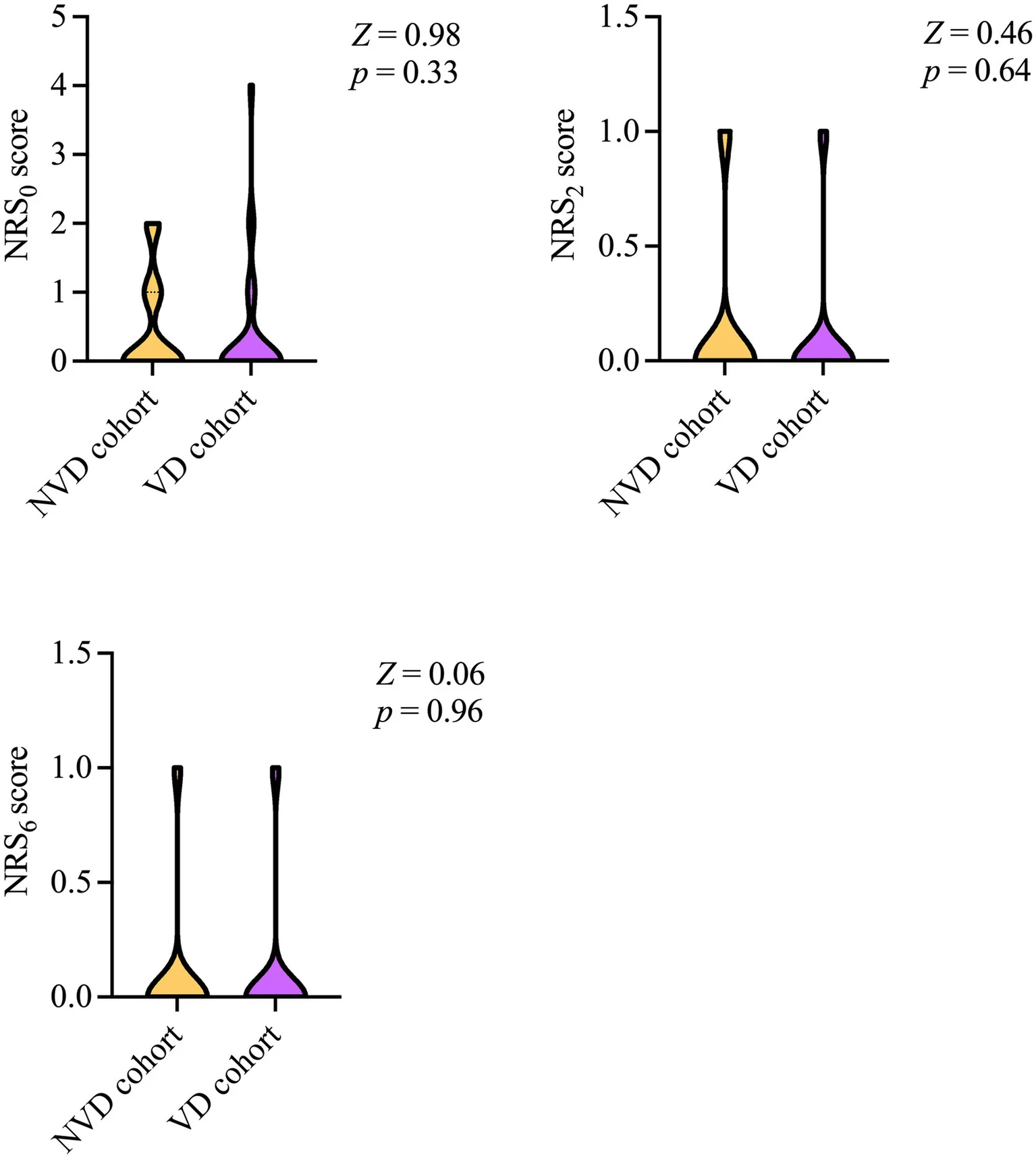
Comparison of NRS scores between NVD and VD cohorts at different time points. Comparison of postoperative pain intensity assessed by NRS between NVD (*n* = 21) and VD (*n* = 30) cohorts. Data are presented across three postoperative time points: NRS_0_ (0 h postoperatively, upon PACU arrival), NRS_2_ (2 h postoperatively), and NRS_6_ (6 h postoperatively). NRS, Numeric Rating Scale; NVD, non-vaginal delivery; PACU, post-anesthesia care unit; VD, vaginal delivery.

No significant differences in NRS scores were observed between the cohorts at 0, 2, and 6 h postoperatively (Figure 5, *p* > 0.05).

## 6 Discussion

This study demonstrates, for the first time, that vaginal delivery history significantly affects the ED_50_ of oliceridine combined with propofol required to suppress body movement during hysteroscopy. Based on this critical clinical variable, we intentionally stratified patients into the NVD cohort and the VD cohort. The results showed that the ED₅₀ in the NVD cohort was 21.603 μg/kg (95% CI: 19.922–23.752), and the ED_95_ was 25.090 μg/kg (95% CI: 23.120–36.414); in the VD cohort, the ED_50_ was 17.816 μg/kg (95% CI: 15.880–19.981) and the ED_95_ was 23.216 μg/kg (95% CI: 20.459–42.365). The significantly higher oliceridine ED_50_ requirement in the NVD cohort (*p* = 0.008) indicates that a history of vaginal delivery is significantly associated with a reduced anesthetic drug demand during hysteroscopic surgery. Rather than acting as a definitive independent predictor, this easily ascertainable obstetric history serves as a highly valuable bedside reference for anesthesiologists to tailor initial dose titrations. The underlying mechanisms may involve cervical structural remodeling (increased compliance) (9) and decreased excitability of mechanosensitive afferent nerves (20) induced by vaginal delivery. These findings not only confirm previous reports that delivery history affects hemodynamics and drug requirements during hysteroscopic surgery under non-intubated general anesthesia (21) but also quantify this influence at the level of specific effective drug doses, establishing for the first time a delivery-history-based dosing reference for hysteroscopy. Therefore, the clinical implication of this study is that anesthesiologists should actively inquire about obstetric history and consider appropriately increasing the oliceridine dose in women without prior vaginal delivery to achieve more optimized intraoperative immobility.

The 21% higher ED_50_ requirement in the NVD cohort than in the VD cohort (21.603 vs. 17.816 μg/kg, *Z* = 2.646, *p* = 0.008) confirms our hypothesis: patients without a history of vaginal delivery require a significantly higher dose of oliceridine than those with such a history to achieve adequate movement suppression during hysteroscopy. This finding carries a clear clinical implication: applying a uniform fixed dose across all patients regardless of obstetric history would risk underdosing patients without prior vaginal delivery while overdosing those with such a history. Incorporating readily available clinical variables, such as vaginal delivery history, refines dose selection beyond standard weight-based starting points, aligning with the broader shift toward personalized anesthesia.

Two recent independent dose-finding trials reported comparable effective doses. Yang et al. (22) used a fixed initial oliceridine dose of 1.5 mg with propofol 2.0–2.5 mg/kg, reporting an ED_50_ of 1.583 mg (95% CI: 1.444–1.857) and an ED₉₅ of 1.848 mg (95% CI: 1.701–4.409). He et al. (23) used an initial oliceridine dose of 1.5 mg and a 1:1.2 dose-adjustment ratio, reporting an ED_50_ of 1.309 mg (95% CI: 1.165–1.477) and an ED₉₅ of 1.675 mg (95% CI: 1.497–2.508); all patients maintained spontaneous breathing without respiratory depression.

Compared with these studies, the key innovations and differences of our study are: (1) Vaginal delivery history stratification: Neither Yang nor He stratified by vaginal delivery history. Our study is the first to introduce this stratification. (2) Dose reporting: Yang and He reported absolute milligram doses without weight standardization; we report weight-standardized doses (μg/kg) and further stratify by vaginal delivery history, enabling more individualized dosing. (3) Propofol co-administration dose: Our propofol induction dose (1.5 mg/kg) was lower than that used by Yang et al. (2.0–2.5 mg/kg) and He et al. (2.5 mg/kg). Given that propofol and oliceridine synergistically suppress movement response, a reduced propofol dose would theoretically require an increased oliceridine dose to maintain equivalent anesthetic depth. Notably, even under our low-dose propofol protocol, the ED₅₀ in the NVD cohort remained 21% higher than that in the VD cohort (21.6 vs. 17.8 μg/kg, *Z* = 2.646, *p* = 0.008).

Zhang Beibei et al. (24) reported that during artificial abortion, the ED₅₀ of oliceridine in patients without vaginal delivery history was 26 μg/kg, compared to 19 μg/kg in those with such a history, suggesting that patients with a history of vaginal delivery may have lower analgesic requirements. Consistent with this observation, our study found that the effective dose of oliceridine may also be correlated with a history of vaginal delivery. This may be related to the higher cervical tension in patients without a vaginal delivery history. The ED_50_ for hysteroscopy in our study was lower than that reported by Zhang Beibei et al. for artificial abortion, which may suggest that the intensity of surgical stimulation during hysteroscopy is lower than that during artificial abortion. *Jia Jia* et al. (25) reported that the ED₅₀ (95% CI) of oliceridine combined with propofol for suppressing the response to endoscope insertion in the adult cohort (18–65 years old) and the elderly cohort (over 65 years old) was 15 (7–23) μg/kg and 12 (7–17) μg/kg, respectively. Cao et al. (26) reported that the ED₅₀ (95% CI) of oliceridine combined with propofol for suppressing movement response during painless gastroscopy was 10.38 (9.02–11.96) μg/kg in females and 12.63 (11.43–13.79) μg/kg in males, using the same modified Dixon sequential method. The ED₅₀ of oliceridine for gastroscopy was even lower, which may imply a relatively weaker stimulation intensity. Taken together, our findings tentatively suggest that the dose requirement of oliceridine may be positively correlated with the degree of surgical trauma, suggesting a potential hierarchy: artificial abortion requires the highest dose, followed by hysteroscopy, and then gastroscopy.

Regarding safety, no serious adverse events occurred. The incidence of propofol injection pain was 15.7% (8/51), lower than the 22.4% reported by Leff et al. (27) without analgesics. Only two patients (3.92% (2/51)) experienced transient hypoxemia (SpO₂ dropped to 90%) due to glossoptosis, which resolved rapidly after jaw thrust, consistent with the mild respiratory effects of oliceridine reported by Simons (28) and Brzezinski (29). In our study, the incidences of postoperative nausea (3.92% (2/51)), vomiting (1.96% (1/51)), and dizziness (1.96% (1/51)) were low. A meta-analysis by Liu et al. (30) showed that compared with morphine, oliceridine significantly reduced the risk of postoperative nausea (RR = 0.55), vomiting (RR = 0.36), dizziness (RR = 0.71), and hypoxemia (RR = 0.52). Beard et al. (31) also reported better gastrointestinal tolerance with oliceridine. The results of these two studies are consistent with the low incidence of adverse reactions observed in our study. These findings are in line with the biased *μ*-opioid pharmacology of oliceridine, which has been shown to reduce typical opioid side effects without loss of analgesia (32). Notably, all patients who experienced nausea or vomiting had a history of motion sickness and an Apfel score of 3, corresponding to a baseline PONV risk as high as 60% (33); thus, the symptoms were not attributable to oliceridine itself. All patients awakened within 7 min without affecting turnover. Only one VD patient reported an NRS score of 4 during recovery, which was relieved by rescue analgesia; all other patients had NRS scores < 3, indicating satisfactory pain control.

### 6.1 Limitations

First, the lack of randomization and absence of blinding may introduce selection and observation biases, which should be considered when interpreting the findings.

Second, although the sample size was methodologically justified for the modified Dixon sequential method, it limits the generalizability of our findings and the assessment of rare adverse events. Future multicenter studies with larger sample sizes are necessary.

Third, our findings apply only to ASA I–II patients; further studies in populations with severe comorbidities or obesity are warranted.

Fourth, the Dixon up-and-down method is known to provide limited precision for ED_95_ estimates (34). Therefore, our reported ED_95_ values should be considered exploratory. To overcome this limitation, we plan to conduct a follow-up study using the biased coin design, which is more appropriate for ED_95_ estimation, and will further stratify by vaginal delivery history to validate and refine the present findings.

Fifth, due to insufficient statistical power for secondary safety endpoints, the safety data should be viewed strictly as descriptive monitoring and not as evidence of equivalence between cohorts.

## 7 Conclusion

The ED_50_ of oliceridine combined with propofol for movement suppression during hysteroscopy was 21.603 μg/kg (95% CI: 19.922–23.752) in the NVD cohort and 17.816 μg/kg (95% CI: 15.880–19.981) in the VD cohort, representing a 21% higher requirement in the NVD cohort (*Z*-test, *p* = 0.008). All adverse events were mild and self-limited, with rapid recovery in both cohorts. These findings suggest that vaginal delivery history is a readily available factor that may inform initial oliceridine dose selection for hysteroscopy.

## Data Availability

The original contributions presented in the study are included in the article/supplementary material, further inquiries can be directed to the corresponding author.
